# Quantitative analysis reveals reciprocal regulations underlying recovery dynamics of thymocytes and thymic environment in mice

**DOI:** 10.1038/s42003-019-0688-8

**Published:** 2019-11-29

**Authors:** Kazumasa B. Kaneko, Ryosuke Tateishi, Takahisa Miyao, Yuki Takakura, Nobuko Akiyama, Ryo Yokota, Taishin Akiyama, Tetsuya J. Kobayashi

**Affiliations:** 1 Graduate School of Engineering, The University of Tokyo, 7-3-1 Hongo, Bunkyo-ku Tokyo, 113-8656 Japan; 2 RIKEN Center for Integrative Medical Sciences, 1-7-22 Suehiro-cho, Tsurumi-ku, Yokohama, Kanagawa 230-0045 Japan; 3 Institute of Industrial Science, The University of Tokyo, 4-6-1 Komaba, Meguro-ku Tokyo, 153-8505 Japan; 4 PREST, Japan Science and Technology Agency (JST), 4-1-8 Honcho Kawaguchi, Saitama, 332-0012 Japan

**Keywords:** Differential equations, Regulatory networks, Computational models, Lymphocyte differentiation, Thymus

## Abstract

Thymic crosstalk, a set of reciprocal regulations between thymocytes and the thymic environment, is relevant for orchestrating appropriate thymocyte development as well as thymic recovery from various exogenous insults. In this work, interactions shaping thymic crosstalk and the resultant dynamics of thymocytes and thymic epithelial cells are inferred based on quantitative analysis and modeling of the recovery dynamics induced by irradiation. The analysis identifies regulatory interactions consistent with known molecular evidence and reveals their dynamic roles in the recovery process. Moreover, the analysis also predicts, and a subsequent experiment verifies, a previously unrecognized regulation of CD4+CD8+ double positive thymocytes which temporarily increases their proliferation rate upon the decrease in their population size. Our model establishes a pivotal step towards the dynamic understanding of thymic crosstalk as a regulatory network system.

## Abstract (web summary)

Kaneko et al. model recovery dynamics of thymocytes and thymic epithelial cells following irradiation. They find that CD4+CD8+ double-positive thymocytes temporarily increase their proliferation rate to compensate for a reduction in population size.

## Introduction

The thymus is an organ responsible for producing a large portion of T cells with appropriate repertoires^1^. However, it is relatively sensitive to insults from stress, viral infection, radiation, and other stimuli^2,3^. While a thymus in a healthy animal can be normally recovered from these damages, a relatively prolonged process of thymic recovery may impair T-cell-mediated immunity due to a reduced replenishment of naïve T-cell repertoire during the recovery period^3,4^.

Sub-lethal dose radiation on mice has been utilized as an experimental model of the thymic regeneration after insults^5,6^. Ionizing irradiation is also broadly used for hematopoietic transplantation and cancer therapy^7,8^, and total body irradiation causes acute thymic injury and slow recovery of thymopoiesis. Several studies have shown that irradiation reduces cellularity, not only of thymocytes but also of thymic epithelial cells (TECs), which are major constituents of the thymic environment^5,9,10^. Because thymopoiesis is supported by interactions between thymocytes and TECs^11^, understanding thymic recovery requires characterization of the reciprocal regulations between thymocytes and TECs.

Concomitantly, various techniques to trace, perturb, and quantify cells involved in these events have enabled us to quantitatively characterize their dynamics^12–15^. By combining mathematical models with such quantitative data, dynamic aspects of thymopoiesis have been distilled into the form of detailed kinetic information, e.g., rates of proliferation, death, and differentiation^12,16^. Mehr et al.^17^ developed the first kinetic model of thymocyte development using ordinary differential equations^18^. Since this seminal work, kinetic models of the thymopoiesis have been progressively refined by considering detailed cellularity and developmental states of the thymocytes, as well as by incorporating different experimental conditions^19–23^.

However, previous works have focused only on thymocytes. Thymic development and thymic recovery are not thymocyte-autonomous but rather are supported by the thymic environment. In the last decade, we have accumulated molecular-biological evidence that the thymic environment itself is homeostatically maintained by thymic crosstalk, bidirectional interactions between the thymocytes and the thymic environment^11,24,25^. Among several cells comprising the thymic environment, cortical and medullary thymic epithelial cells (cTECs and mTECs) play integral roles in inducing and controlling proliferation, apoptosis, and lineage commitments of thymocytes^11,26–30^. Thymocytes also regulate TECs by modulating their maturation and proliferations^5,31–33^. Despite the evident relevance and importance of thymic crosstalk for the thymopoiesis and the thymic recovery, kinetic aspects of the reciprocal regulations between the thymocytes and the TECs have not yet been clarified.

In this work, we investigate the joint dynamics of thymocytes and TECs by combining a mathematical model with a quantitative measurement of the number of thymocytes and TECs during recovery after irradiation. Recovery dynamics are reproduced by our mathematical model, in which we identified reciprocal interactions between thymocytes and TECs that are relevant for recovery and consistent with thymic crosstalk. Furthermore, we demonstrate that the model provides an explanation for the mechanism of the dynamical change in population size. Particularly, our model predicts, and a subsequent experiment verifies, a previously unrecognized regulation of CD4+CD8+ double-positive (DP) thymocytes, which temporarily increases their proliferation rate upon the decrease in their population size.

## Result

### Quantification of recovery dynamics of thymocytes and TECs

**Fig. 1:**
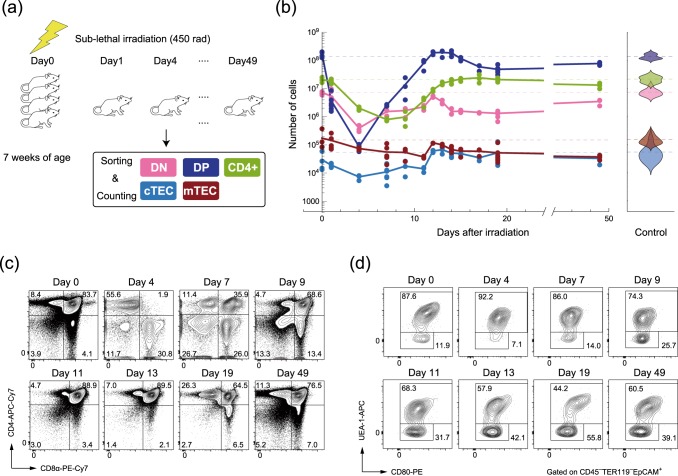
Recovery dynamics of thymocytes and TECs after sub-lethal irradiation. **a** A schematic diagram of the perturbation experiment. **b** The left panel shows trajectories of the counts of thymocytes (DN: pink, DP: blue, SP4: light green) and TECs (cTEC: cyan, mTEC: brown) after irradiation. Points correspond to the experimental cell counts, and the solid curves are linear interpolations of the average counts at each time point. The numbers of samples at each time point are shown in Table 1. The right panel shows violin plots of the numbers of thymocytes and TECs without perturbation (*n* = 15 for thymocytes, *n* = 16 for TECs). **c** Typical flow cytometric profiles of the thymocytes after the sub-lethal dose radiation. Thymocytes were analyzed by staining with anti-CD4 and anti-CD8α. Percentage of each fraction is shown in the panels. **d** Typical flow cytometric profiles of TECs after the sub-lethal dose radiation. TECs (EpCAM^+^CD45^−^TER119^−^) were analyzed by staining with a combination of UEA-1 lectin and anti-CD80. Percentages of UEA-1^+^ cells (mTECs) and UEA-1^–^ cells (cTECs) are shown in the panels.

To quantitatively investigate the kinetic relationship between thymocytes and TECs as well as the establishment of thymic recovery, we artificially perturbed populations of thymocytes and TECs in thymi by using sub-lethal 4.5 Gy irradiation, and measured the dynamic changes in their population sizes over 3 weeks following irradiation (Fig. 1a and Table 1). Figure 1b summarizes the changes in cell numbers, which were sorted based on conventional markers of thymocytes (Fig. 1c and Supplementary Fig. 1a) and TECs (Fig. 1d and Supplementary Fig. 1b). Figure 1b shows that all types of investigated thymocytes and TECs decreased exponentially at different rates immediately after the irradiation. Then, both thymocytes and TECs started recovering within 10 days at the longest; the CD4−CD8− double-negative (DN) thymocytes and the cTECs began recovery within 5 days, whereas the CD4+CD8− single-positive (SP) thymocytes and the mTECs required longer intervals, reflecting the temporal order of the thymocyte development from DN to CD4+ SP (SP4) cells through interactions from cTECs to mTECs.

Upon recovery, the population sizes of all but the SP cells peaked around 15 days, and eventually returned to stationary numbers, which were almost equivalent to or at least half of the original population sizes before irradiation. Such overshooting behaviors suggest that the numbers of thymocytes and TECs are dynamically and mutually regulated via reciprocal interactions (Table 1).

**Table 1:** The numbers of samples at each time point after irradiation.

Days after irradiation	0	1	4	7	9	11	12	13	14	15	17	19	49
Number of samples (thymocytes)	4	6	3	3	3	3	2	3	3	3	3	6	3
Number of samples (TECs)	4	6	3	6	3	3	2	3	6	3	3	6	3

### Mathematical model can reproduce recovery dynamics

To infer regulatory interactions behind the dynamics, we constructed a mathematical model for the population dynamics of the thymocytes and the TECs using ordinary differential equations, which explicitly include five cell types:

$i \in C = \{ {\mathrm{DN}},{\mathrm{DP}},{\mathrm{SP}}4,{\mathrm{cTEC}},{\mathrm{mTEC}}\}$. To account for the acute influence of irradiation on the cells, the total number of the cell type *i* at time *t* (day), $n_i^{{\mathrm{tot}}}(t)$ is decomposed into two parts; $n_i^{\mathrm{x}}(t)$ represents exponentially dying cells by the irradiation and *n*_*i*_(*t*) represents cells that survived or were newly generated after irradiation. $n_i^{\mathrm{x}}(t)$ is assumed to decrease exponentially at a constant rate, *ω*_*i*_ (day^−1^), as $n_i^{\mathrm{x}}\left( t \right) = n_i^{{\mathrm{tot}}}\left( 0 \right)\left( {1 - p_i} \right)e^{ - \omega _it}$, where *p*_*i*_ is the proportion of survived cells after irradiation; we modeled the dynamics of *n*_*i*_(*t*) with ordinary differential equations. Therefore, the total number of cell type *i*, $n_i^{{\mathrm{tot}}}$, which we observed experimentally, is described as $n_i^{{\mathrm{tot}}}\left( t \right) = n_i^{\mathrm{x}}\left( t \right) + n_i(t)$.

The temporal change in *n*_*i*_(*t*) is driven by the imbalance among influx, proliferation, death, and outflux of the type *i* cells, each of which depends on the numbers of other cells ${\mathbf{n}}\left( t \right): = \left[ {n_{{\mathrm{DN}}}\left( t \right),n_{{\mathrm{DP}}}\left( t \right),n_{{\mathrm{SP}}4}\left( t \right),n_{{\mathrm{cTEC}}}\left( t \right),n_{{\mathrm{mTEC}}}\left( t \right)} \right]^T$, where *T* denotes transpose. While the influx may be independent of the number of type *i* cells, the other should, in nature, depend on the number of existing type *i* cells, *n*_*i*_(*t*). This allows us to generally represent the ordinary differential equations for *n*_*i*_(*t*) as

$$\frac{{{\mathrm{d}}n_i(t)}}{{{\mathrm{d}}t}} = \phi _i\left( {{\mathbf{n}}\left( t \right)} \right) + f_i\left( {{\mathbf{n}}\left( t \right)} \right)n_i\left( t \right),$$

where the influx should be non-negative, $\phi _i\left( {{\mathbf{n}}\left( t \right)} \right) \ge 0$, whereas the marginalized rate of proliferation, death, and outflux, $f_i({\mathbf{n}}(t))$, can be either positive or negative. The actual value of $f_i({\mathbf{n}}\left( t \right))$ is determined by the balance among proliferation, cell death, and outflux of type *i* cells. To obtain a minimal model with minimal complexity, we assume that both $\phi _i\left( {{\mathbf{n}}\left( t \right)} \right)$ and $f_i({\mathbf{n}}(t))$ are at most linear with respect to **n**(*t*) with possible constant time delays. Therefore, our ordinary differential equation model as a whole has, at most, quadratic nonlinearity. Considering reproducibility of the recovery dynamics after the irradiation and consistency with previously known molecular evidence, we obtained the whole model described as:

1 $$\frac{{{\mathrm{d}}n_{{\mathrm{DN}}}\left( t \right)}}{{{\mathrm{d}}t}} =	 \; \phi _1 + \left( {\delta _1 - \mu _1n_{{\mathrm{cTEC}}}\left( t \right)} \right)n_{{\mathrm{DN}}}(t),\\ \frac{{{\mathrm{d}}n_{{\mathrm{DP}}}(t)}}{{{\mathrm{d}}t}} =	 \; \mu _1r_1n_{{\mathrm{cTEC}}}\left( t \right)n_{{\mathrm{DN}}}\left( t \right)\\ 	+ \left\{ {\theta _2\left( {1 - \frac{{n_{{\mathrm{DP}}}\left( t \right)}}{{K_2}}} \right) - \mu _2n_{{\mathrm{cTEC}}}\left( {t - \tau _2} \right)} \right\}n_{{\mathrm{DP}}}(t),\\ \frac{{{\mathrm{d}}n_{{\mathrm{cTEC}}}(t)}}{{{\mathrm{d}}t}} =	 \; \phi _{\mathrm{c}} + \left( { - \delta _{\mathrm{c}} + \mu _{\mathrm{c}}n_{{\mathrm{DN}}}\left( t \right)} \right)n_{{\mathrm{cTEC}}}(t),\\ \frac{{{\mathrm{d}}n_{{\mathrm{SP}}4}(t)}}{{{\mathrm{d}}t}} = 	\; \mu _2r_{24}n_{{\mathrm{cTEC}}}\left( {t - \tau _2} \right)n_{{\mathrm{DP}}}\left( t \right) - \mu _4n_{{\mathrm{mTEC}}}\left( t \right)n_{{\mathrm{SP}}4}\left( t \right),\\ \frac{{{\mathrm{d}}n_{{\mathrm{mTEC}}}(t)}}{{{\mathrm{d}}t}} =	 \; \phi _{\mathrm{m}} + \phi _{{\mathrm{m}}4}n_{{\mathrm{SP}}4}\left( t \right),\\ 	+ \left\{ {r_{\mathrm{m}}\left( {1 - \frac{{n_{{\mathrm{mTEC}}}(t)}}{{K_{\mathrm{m}}}}} \right) - \gamma _{{\mathrm{mp}}}n_{{\mathrm{DP}}}\left( {t - \tau _{\mathrm{m}}} \right)} \right\}n_{{\mathrm{mTEC}}}(t),$$

a diagrammatic representation of which is shown in Fig. 2a. Based on this model with candidate parameter values as the initial condition, we conducted a nonlinear least square estimation of the whole parameter values in Eq. (1), $\{ n_i^{{\mathrm{tot}}}\left( 0 \right)\} _{i \in C}$, $\{ \omega _i\} _{i \in C}$, and $\left\{ {p_i} \right\}_{i \in C}$ so that the whole model can reproduce all the experimental data at once (Fig. 2b and Table 2). As shown in Fig. 2b, our model, Eq. (1), nicely reproduced the experimentally observed recovery, demonstrating that the interactions depicted in Fig. 2a sufficiently account for the dynamics. Moreover, to reevaluate the importance and statistical confidence of several parameters, we statistically estimated the potential variability of the estimated values by conducting a bootstrap parameter estimation (Figs. 2c and 3, and Supplementary Table 1). As shown in Fig. 3, most parameter values statistically fluctuate around single peak, whereas a few parameters, e.g., the influx rate of DN, *ϕ*_1_, have multiple peaks in their estimates.

**Fig. 2:**
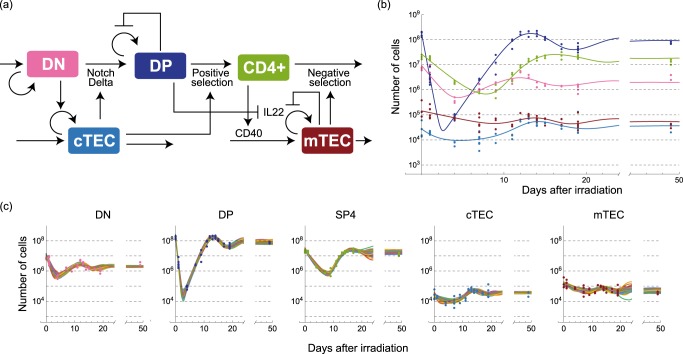
Schematic diagram and trajectories of the mathematical model inferred from the quantitative data. **a** A schematic diagram of the intercellular interactions inferred from the experimental data and represented by Eq. (1). **b** Trajectories of the numbers of thymocytes and TECs obtained by simulating Eq. (1) with the optimally fitted parameter set. The curves represent simulated trajectories, and the points represent the same experimental data as Fig. 1b. Cell types are designated by the color codes which are defined in **a**. **c** Trajectories obtained by the bootstrap parameter estimation. Trajectories in different panels with the same color correspond to a simulation with a parameter set estimated from a bootstrapped sample. The trajectories of 100 randomly selected samples are shown in the panels. The points represent the same experimental data as Fig. 1b.

**Table 2:** A comparison of the estimated kinetic rates with those from previous studies.

Term	Value	CI	Previous study
Inflow rate to DN [cells day^−1^] (estimated by the coarse-grained model)	3.3 × 10^4^	[3.3 × 10^3^, 6.6 × 10^4^]	2.0 × 10^4^ (^19^), 1.3 × 10^4^ (^43^)
Inflow rate to DN (cells day^−1^) (estimated by the detailed model)	6.61 × 10^1^	N/A	10^1^ ~ 2 × 10^2^ (^36^), 4.9 × 10^1^ (^20^)
DN apparent proliferation rate (day^−1^)	1.3 × 10^−1^	[−2.2 × 10^−2^, 3.3 × 10^−1^]	2.3 × 10^−1^ (^19^), 6.2 × 10^−4^ (^43^) 3.6 × 10^−1^ (^20^)
DN differentiation rate (day^−1^)	1.4 × 10^−1^	[5.7 × 10^−6^, 3.5 × 10^−1^]	2.4 × 10^−1^ (^19^), 2.8 × 10^−2^ (^43^) 3.4 × 10^−1^ (^20^), 4.5 × 10^−1^ (^42^)
DN residence time (hour)	1.7 × 10^2^	[6.9 × 10^1^, 4.3 × 10^6^]	4.2 × 10^2^ (^19^), 3.5 × 10^2^ (^20^)
DP apparent proliferation rate (day^−1^)	1.0 × 10^−1^	[5.8 × 10^−2^, 2.5 × 10^−1^]	1.5 × 10^−2^ (^19^), −3.7 × 10^−1^ (^21^) −1.6 × 10^−1^ (^42^), −9.0 × 10^−3^ (^43^)
DP differentiation rate to SP4 (day^−1^)	1.1 × 10^−1^	[6.0 × 10^−2^, 2.5 × 10^−1^]	2.1 × 10^−2^ (^19^), 1.2 × 10^−2^ (^21^) 3.0 × 10^−2^ (^42^), 9.9 × 10^−2^ (^43^)
DP residence time (hour)	2.3 × 10^2^	[9.5 × 10^1^, 4.0 × 10^2^]	9.4 × 10^1^ (^19^), 7.6 × 10^1^ (^21^), 1.2 × 10^2^ (^22^)
Proportion of DP to SP4 in DP export (%)	10	[4.9, 30]	6.0 (^19^), 0.016 (^21^), 8.1 (^42^), 65 (^43^)
SP4 apparent export rate (day^−1^)	5.2 × 10^−1^	[2.7 × 10^−1^, 1.2 × 10^0^]	2.0 × 10^−2^ (^19^), 9.0 × 10^−2^ (^21^) 1.4 × 10^−1^ (^42^), 1.7 × 10^−1^ (^43^)

(CI: confidence interval) The value of each term is estimated in our model by the following equations of the parameters evaluated at the steady state $n_i^ \ast$

Inflow rate to DN thymocytes: *ϕ*_1_

DN apparent proliferation rate: $\delta _1 - \mu _1\left( {1 - r_1} \right)n_{{\rm{cTEC}}}^ \ast$

DN differentiation rate: $\mu _1r_1n_{{\rm{cTEC}}}^ \ast$

DN residence time: $24/(\mu _1r_1n_{{\rm{cTEC}}}^ \ast )$

DP apparent proliferation rate: $\theta _2(1 - n_{{\rm{DP}}}^ \ast /K_2) - \left( {1 - r_{24}} \right)\mu _2n_{{\rm{cTEC}}}^ \ast$

DP differentiation rate to SP4: $r_{24}\mu _2n_{{\rm{cTEC}}}^ \ast$

DP residence time: $24/(r_{24}\mu _2n_{{\rm{cTEC}}}^ \ast )$

Proportion of DP to SP4 in DP export: *r*_24_

SP4 apparent export rate: $\mu _4n_{{\rm{mTEC}}}^ \ast$

We note that our point estimate of the DP residence time 230 h may be an overestimate, although the previous estimates overlap the statistically confident range of the values obtained by our bootstrap analysis. This is because the residence time was estimated only from the output flux rate, due to the fact that the apoptosis rate cannot be estimated in our model

**Fig. 3:**
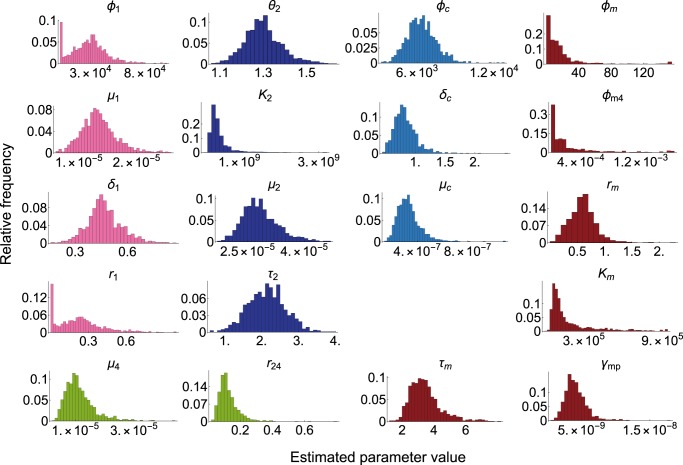
Variations of parameters estimated by bootstrap parameter estimation. The color of each histogram of a parameter designates the related cell type in Fig. 2a to that parameter. The variations of the other parameters and pairwise scatter plots of the estimated values are also shown in Supplementary Figs. 2 and 3, respectively.

### DN thymocytes and cTECs form a negative feedback

Our estimated model indicates that DN thymocytes and cTECs form a negative feedback. DN cells marginally work to increase the number of cTECs because *μ*_c_ in Eq. (1) is positive, whereas cTECs effectively inhibit the increase in DN cells because −*μ*_1_ in Eq. (1) is negative (Fig. 2a). This negative feedback is the source of the overshooting behaviors in the recovery dynamics, and can account for slower onset of cTECs recovery, which lagged a few days behind DN cells.

These interactions inferred from the quantitative recovery data are also consistent with previously identified molecular evidence. On one hand, the positive interaction from DN thymocytes to cTECs may be interpreted as induction of cTEC proliferation by DN cells, evidenced by the fact that the number of mature cTECs decreases if DN differentiation is blocked at early stages^31,34^. On the other hand, our model suggests that cTECs work to decrease the number of DN cells. This negative interaction is a marginal effect of induced cell death, induced differentiation from the DN to the DP stages, and inhibition of DN proliferation by cTECs. This negative regulation of DN cells by cTECs is consistent with the lineage commitment of DN cells to the DP stage mediated by cTECs in Notch1-Delta-like4-dependent manner^27,28^. It should be noted, however, that our model does not exclude other possibilities of additional molecular interactions, as long as their marginal influences are consistent with the diagram in Fig. 2a.

**Fig. 4:**
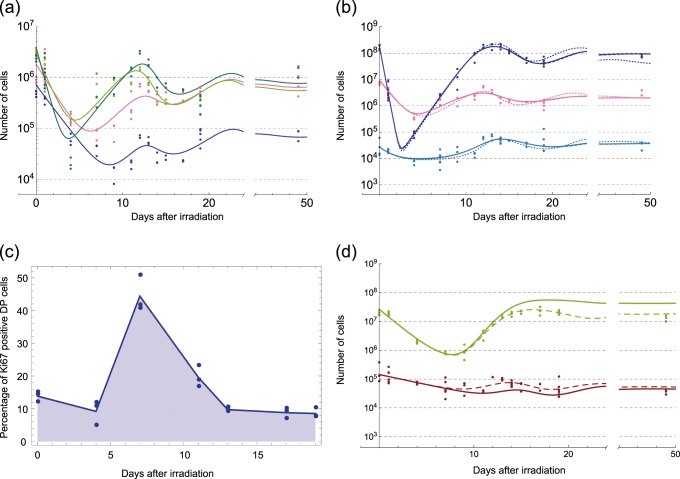
Detailed analysis of the proposed mathematical model Eq. (1). **a** Dynamics of DN subpopulations obtained experimentally with the corresponding fitted trajectories of the detailed model. DN1: pink, DN2: blue, DN3: light green, DN4: green. **b** A comparison of the trajectories obtained by the detailed model (dotted line) with those of the coarse-grained model for high (solid line) and low (broken line) DN influx rates. The solid and broken lines are almost perfectly overlapped in this panel. The colors represent cell types; DN: pink, DP: blue, cTEC: cyan. **c** Validation of the model prediction by a proliferation assay of DP cells. Percentages of Ki67-positive DP cells are obtained at 0, 4, 11, 13,17, and 19 days after irradiation. Points represent experimental cell counts, and shaded lines represent linear interpolations of the average counts (*n* = 3 at each time point). **d** In silico evaluation of the impact from the disturbed crosstalk between SP4 thymocytes (light green) and mTECs (brown). Thick solid curves are simulated trajectories of SP4 thymocytes and mTECs with parameter values mimicking the experimental condition in ref. ^33^, *γ*_4_ = 5.0 × 10^−6^ and *ϕ*_*m*4_ = 0. The thin broken curves are those obtained with the optimal parameter values used in Fig. 2b for comparison.

To further analyze the consistency of our model with the underlying dynamics of DN subpopulations (DN1, DN2, DN3, and DN4), we additionally quantified the dynamics of these populations after irradiation (Supplementary Fig. 4). We also modified Eq. (1) (denoted here as a coarse-grained model) to include DN subpopulations (denoted as a detailed model and shown in Methods), the parameter values of which were similarly estimated. As demonstrated in Fig. 4a, b, the detailed model reproduces the dynamics of the DN subpopulations (Fig. 4a) with only small deviation from the coarse-grained model in which DN subpopulations are lumped together (Fig. 4b). We should mention that our estimates of DN1 and DN2 subpopulations can be overestimates because an additional cell surface marker, CD117, is required to exclude non-T-lineage fractions^35^. This overestimate, however, has little effect on the inferred dynamics of the total DN population (Fig. 4b), because the major fraction of DN cells consists of DN3 and DN4 cells. The estimated parameter values were also consistent with those of the coarse-grained model except for the DN1 influx rate *ϕ*_1_ estimate, which was much smaller than that of coarse-grained model. Because the peak other than that around the optimal value in the bootstrap estimate of *ϕ*_1_ was at the lower bound of its estimation range (Fig. 3), a value smaller than the lower bound may also reproduce the same recovery dynamics. To verify whether the estimate of *ϕ*_1_ in the detailed model is also consistent with the coarse-grained model, we simulated coarse-grained model by replacing the value of *ϕ*_1_ in the model with the estimate from the detailed model. As shown in Fig. 4b, the trajectories were almost unaffected by this replacement. Moreover, from the biological viewpoint that the number of the influx DN progenitors is quite small^36^, this value of *ϕ*_1_ is also reasonable. Altogether, analysis of the detailed model revealed that the smaller value of *ϕ*_1_, which cannot be selected only from the analysis of the coarse-grained model, is more relevant.

### DP recovery by temporal increase in proliferation rate

The kinetic component characteristic to the DP dynamics is its much faster recovery compared to DN cells (Fig. 1b), which strongly suggests that the DP recovery is achieved by self-proliferation rather than by the influx from the DN population. However, evidence about the self-proliferation ability and speed of DP cells is inconsistent and may depend on strains^36,37^; some studies showed that DP cells proliferate little^22,38^ while others have suggested that DP cells can proliferate faster than other types of thymocytes^37,39^. Our model coordinates these properties with auto-inhibitory regulation of the DP proliferation, represented by the logistic term $\theta _2(1 - n_{{\mathrm{DP}}}(t)/K_2)$ in Eq. (1), which can realize fast proliferation during the recovery period and slowdown at the steady state. Nevertheless, such auto-inhibitory regulation in DP proliferation has not yet been reported.

To experimentally verify this prediction by our model, we estimated the fraction of proliferating DP cells under the same condition as in Fig. 1a by staining the DP population with proliferation marker Ki67 (Fig. 4c). We observed that the fraction of the proliferating DP cells transiently increased and peaked at day 7 after irradiation, coinciding perfectly with the timing of exponential increase in DP cells during recovery. Self-proliferation ceased when the number of the DP cells recovered to the normal population size before the irradiation. This result strongly supports that the proliferation rate of DP cells is inhibited by total population size to maintain homeostasis. Further, this autoregulatory mechanism is consistent with the previous observations that DP cells proliferate little when their numbers are at the steady state^38^.

While the autoregulatory proliferation of DP cells is necessary for reproducing fast recovery, it cannot solely account for the overshooting behavior of DP cells, which suggests that other cells regulate DP cells. Supported by well-established evidence that cTECs engage in positive selection of DP cells, our model includes a negative influence of cTECs to DP cells with a time delay, which can nicely reproduce the overshoot of DP cell count. This negative interaction with a time delay can be interpreted as the marginal effect of an induced apoptosis of DP cells with non-functional T cell receptors (TCRs) and the differentiation of DP cells into SP cells upon apoptosis rescue. The existence of the time delay may be interpreted by the sequential and multiple interactions of DP cells with cTECs that are required for positive selection.

Our model estimates that the stable rate of DP cells to differentiate into CD4 SP cells, $r_{24}\mu _2n_{{\mathrm{cTEC}}}^ \ast$, ranges from 6.0 × 10^−2^ to 2.5 × 10^−1^ (day^−1^), overlapping the range of the previous estimates of 1.2 × 10^−2^ to 9.9 × 10^−2^ (day^−1^) (Table 2). The estimated value of *r*_24_ varied from 4.9% to 30%, within the range of the previous estimates that 0.02–65% of DP cells survive and differentiate into CD4 SP via positive and negative selections (Table 2). This result supports the interpretation that *r*_24_ is the fraction of rescued DP cells that differentiated into CD4 SP, and the remaining fraction 1 − *r*_24_ of DP cells undergoes apoptosis. However, we should note that an apoptosis rate cannot be directly estimated solely by population size dynamics. This may be the major reason why the estimated fraction of the rescued DP cells varies in our and previous studies.

### DP and CD4 SP thymocytes incoherently regulate mTEC recovery

Compared with other thymocytes and TECs, CD4 SP cells recovered much slower, with less pronounced overshooting (Fig. 1b). This slow recovery of CD4 SP cells is consistent with their lack of proliferation capacity^13,37^, which leads to prolonged recovery. The CD4 SP dynamics can be reproduced by assuming no proliferation and mTEC-dependent death and outflux $- \mu _4n_{{\mathrm{mTEC}}}(t)$, which may represent the negative selection of SP cells by mTECs (Fig. 2a).

**Fig. 5:**
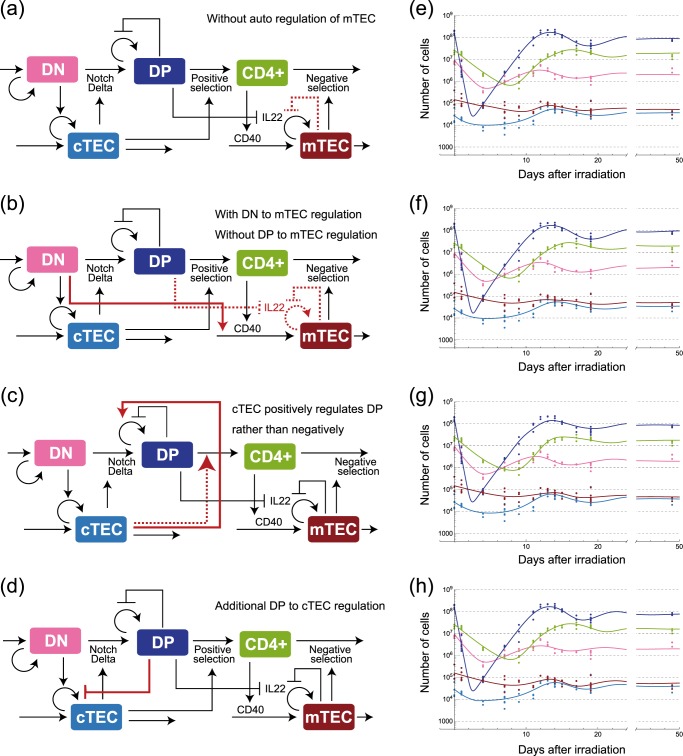
Possible regulatory mechanisms that are capable of reproducing the recovery dynamics of the data, but that are biologically less relevant than the proposed model shown in Fig. 2a. Differences between each model and the one shown in Fig. 2a are designated by red solid lines if additionally included or red broken lines if excluded. The equations corresponding to the models are shown in Methods. The model in **a** excludes the autoinhibitory regulation of mTECs. The model in **b** includes an interaction from DN cells to mTECs influx instead of the inhibition from DP cells. The model in **c** assumes that cTECs promote DP cell proliferation, rather than inducing DP cell differentiation or cell death. The model in **d** includes an inhibitory regulation of cTECs from DP cells similar to that of mTECs. **e**, **f**, **g**, and **h** show corresponding trajectories of the models in **a**, **b**, **c**, and **d**.

In contrast, the mTEC recovery was initiated almost concurrently with that of cTEC (Fig. 1b). While interactions with CD4 SP cells have been proven essential for the maturation of mTECs^40^, the prolonged CD4 SP recovery is insufficient for reproducing earlier onset of mTEC recovery. Our model incorporates an auto-inhibitory regulation of mTEC proliferation $r_{\mathrm{m}}(1 - n_{{\mathrm{mTEC}}}\left( t \right)/K_{\mathrm{m}})$ and its negative regulation by DP cells with a time delay $- \gamma _{{\mathrm{mp}}}n_{{\mathrm{DP}}}(t - \tau _{\mathrm{m}})$ as in Eq. (1). The auto-inhibitory regulation is necessary because without it, we obtained biologically inconsistent parameter values in mTEC dynamics (Fig. 5a, e). The negative regulation by DP cells is also responsible for mTEC overshooting. Preceding experimental investigations^5,41^ support these mechanisms. Metzger et al. reported that the percentage of Ki67hi mTECs increases only after the depletion of mTECs^41^, suggesting auto-inhibitory regulation. Based on a depletion experiment of DP cells, Dudakov et al. suggested that DP cells negatively regulated TEC proliferation in an IL22-dependent manner^5^. However, the DP-dependent regulation was not the sole interaction that could explain the early onset of mTEC recovery. We also found that a DN-dependent regulation could reproduce it (Fig. 5b, f). However, this possibility was excluded in our model because we lack molecular evidence supporting the long-range interaction from DN cells to mTECs, which reside in spatially segregated areas of a thymus.

Along with regulated proliferation, our model assumes reciprocal regulations between mTECs and CD4 SP cells to account for evidence that mTEC maturation is also related to CD4 SP cells. According to Williams et al.^33^, mTECs express ligands CD80 and CD86 and a receptor, CD40; the corresponding ligand and receptor of CD4 SP cells are mainly CD28 and CD40L. A knockout of CD80, CD86, and CD40 was shown to decrease the number of mTECs and double the number of CD4 SP cells. We substituted smaller values of *μ*_4_ and *ϕ*_*m*4_ than the estimated values into our model to reproduce the experiment in ref. ^33^ by assuming that the knockout of CD80, CD86, and CD40 corresponds to this substitution. The result qualitatively reproduced the knockout mutant result in ref. ^33^; the stable number of CD4 SP cells doubled whereas the number of mTECs was decreased, as shown in Fig. 4d.

## Discussion

From quantitative time-series data of thymocytes and TECs recoveries after sub-lethal X-ray irradiation, we constructed a mathematical model for the recovery dynamics of thymocytes and TECs. The model reproduces the transient dynamics of the cell population sizes fairly well, and most of the interactions identified by the modeling are consistent with known molecular evidence.

Since previous modeling works on quantitative characterizations of thymocyte development focused only on thymocyte dynamics, our work, which additionally includes both the dynamics of and the interactions with TECs, can be viewed as an extension of those works^17,19–21,42,43^. We validated that the estimated parameter values of thymocytes in our model are mostly consistent with those estimated in previous works (Table 2). Few parameter value mismatches may also be attributed to differences in the experimental setting and conditions. However, it should be noted that we compared the apparent proliferation rates of our model to previous estimates, which are the marginal rates of population size change owing to the imbalance between proliferation and apoptosis because pure rates of proliferation or apoptosis cannot be estimated from our model. To reveal dynamical change in apoptosis rates, we must develop a new approach that combines our reciprocal regulation model with experimental methods that can directly quantify thymocyte proliferation, apoptosis, and differentiation^14,44^. Further, the parameters of TECs are the first to be estimated by modeling, and should be verified by independent research. Particularly, damage caused by irradiation can affect the thymic tissue structure, which may result in a systematic bias when counting TECs^45,46^. While this systematic bias is effectively absorbed in our model by parameters scaling, this potential scaling must be considered when we compare our TEC parameter value estimates with others. Moreover, to assess this problem more carefully, we must develop a new image analysis method that can accurately detect and count cells in 3D tissue images obtained by advanced imaging and tissue clearing^47,48^.

Thymic crosstalk includes various signaling pathways, indicating complex regulations behind the population size control of thymocytes and TECs. Because of this complexity, our model may contain missing interactions or possibly different regulations, some of which were tested during our model identification process. Such possibilities cannot be excluded by the limited amount of data alone; therefore, we employed previously obtained molecular-biological evidence and quantitative estimates to evaluate the possible models.

For example, cTECs rescue DP thymocytes from apoptosis via positive selection, which leads to increase in the DP population size. Concurrently, positive selection also induces differentiation of thymocytes from the DP stage to the SP stage, causing DP population size to decrease. These contradicting interactions introduce the possibility that cTECs increase the DP thymocyte population size, rather than decreasing it as assumed in our model. We examined this possibility by introducing the increasing effect of the DP population size by cTECs and concluded that the decreasing effect assumed by our model is more valid because the model with the increasing effect resulted in much higher parameter values than expected based on the previous works (Fig. 5c, g).

We also investigated a model in which DP thymocytes contribute to the recovery of both mTECs and cTECs^5^. We found that the estimated parameter for the interaction from DP cells to cTECs was almost 0 (Fig. 5d, h), which does not support a major contribution of DP cells to cTECs recovery under our experimental condition.

Our model can explain the mechanisms by which specific dynamics appear in recovery dynamics and their potential biological functions; overshoots of DN thymocytes and cTECs may originate from negative feedback between them and may contribute to prompt recovery from various perturbations affecting thymocyte and TEC numbers. Similarly, the disinhibition of DP proliferation upon DP population size decrease facilitates the swift recovery of DP cells, which could not be achieved solely by the influx of DN cells, as they have a much smaller population size than DP cells. Our model provides an integrative view of thymic crosstalk as a regulatory network and serves as a starting point for comprehensively understanding homeostasis in thymic development.

However, our model still has room for future improvement by accommodating more detailed information on the cellularity of the thymic resident cells, such as B cells, dendritic cells, and thymic endothelial cells. These cells may have different roles in the dynamic regulation of thymic homeostasis than thymocytes and TECs, although we did not explicitly include them by presuming that their effects to the number of thymocytes or TECs are relatively small or constant, which was implicitly modeled by the constant parameters in our model. Actually, BMP4 production by endothelial cells after irradiation, which can contribute to TECs recovery, was reported constant when normalized by the size of thymus^49^. Explicitly incorporating these cells may be crucial to extending our model to other experimental settings as well as for deriving a more integrative and comprehensive model of thymic development and homeostasis. Among others, the repertoires of thymocytes are particularly relevant. TECs are responsible for controlling the number of thymocytes as well as for selecting thymocytes with appropriate repertoires. An upcoming challenge may be integratively modeling and analyzing thymic homeostasis, both in cell numbers and repertoires by combining quantitative measurement and high-throughput sequencing^50^.

## Methods

### Ethics statement

Animals used in the present study were maintained in accordance with the “Guiding Principles for Care and Use of Animals in the Field of Physiological Science” set by the Physiological Society of Japan. All animal experiments were approved by the Animal Research Committees of RIKEN.

### X-ray irradiation and flow cytometory with mice

Balb/cA mice were purchased from CLEA Japan. Female mice (7 weeks old) received X-ray radiation (4.5 Gy). At each sampling point after irradiation, the mice were sacrificed and their thymi were used for a flow cytometric analysis. Each thymus was cut and gently agitated in 2 ml of RPMI-1640 (Sigma-Aldrich, St. Louis, MO, USA) to release thymocytes for the flow cytometric analysis. The days of measurement and the number of sampled mice are shown in Fig. 1a. The remaining thymic tissue was digested using Liberase in RPMI1640 (Wako) at 37 °C for 30 min. The thymic stroma-rich fraction was analyzed by flow cytometry to detect TEC populations. For flow cytometric staining, cells were pre-treated with anti-CD16 and CD32 (Biolegend) for 20 min and subsequently stained with fluorescence-labeled antibodies in phosphate buffered saline containing 3% fetal bovine serum. The stained cells were analyzed using Canto II (BD). The total thymic cell numbers were determined by the sum of cells in the thymic stroma-rich fraction and the thymocyte fraction. TECs were defined as CD45-TER119-EpCAM+ cells. mTECs and cTECs were separated with UEA-1 staining. For DN thymocyte staining, the lineage negative cell fraction was separated by staining with CD25 and CD44 antibodies. Since the DN population contains other minor cell populations such as dendritic cells, the number of cells from these fractions was subtracted from the number of DN cells in the mathematical modeling based on the average percentage of these cells (16.6%) in the DN fraction under steady conditions (Supplementary Fig. 1a). PECy7-anti-CD4 (clone RAM4-4, used as ×200 dilution), FITC-anti-CD4 (clone RAM4-4, ×200 dilution), APCCy7-anti-CD8 (clone 53-6.7, ×200), APCCy7-anti-CD45 (clone 30 F-11, ×200), APCCy7-anti-TER119 (clone TER-119, ×200), FITC-anti-EpCAM (BioLegend, clone G8.8, ×400), PE-anti-CD80(clone 16-10A1, ×400), Biotin-anti-mouse Ly-6G/Ly-6C(Gr-1) (×400), Biotin anti-mouse/human CD45R/B220 (×400), Biotin anti-mouse TER-119/Erythroid cells (clone TER-119, ×400), Biotin conjugated anti-mouse CD11b (×400), PE anti-mouse/human CD44 (clone IM7, ×400), APC anti-mouse CD25 (clonePC61, ×400), Streptavidin PE-Cyanine7 (×400), and Streptavidin-PECy7 (×400) were purchased from Biolegnd. UEA-biotin (×400) was from Vector laboratories (Burlingame, CA).

### Estimation of the fraction of proliferating DP cells

Thymocytes were pre-treated with anti-CD16 and CD32 (Biolegend) and subsequently stained with anti-CD4 and anti-CD8 antibodies in phosphate buffered saline containing 3% FBS. The cells were fixed and permeabilized with Foxp3/Transcription Factor Staining Buffer Set (eBioscience) according to the manufacturer’s protocol. After fixation and permeabilization, the cells were stained with a PE-labeled anti-Ki67 antibody (Biolegend) and subsequently analyzed by Canto II (BD).

### Statistics and reproducibility

For each experimental condition, we measured the numbers of thymocytes and TECs from at least two mice, independently. The variations of parameter values for the mathematical model were analyzed by the bootstrap method described in the section “Confidence interval by bootstrap”.

### Mathematical modeling of thymocyte and TEC dynamics

We assume that the total number of the type *i* cells, $n_i^{{\mathrm{tot}}}$, is the sum of cells dying by irradiation $n_i^{\mathrm{x}}$ and survived or newly generated cells *n*_*i*_:

$$n_i^{{\mathrm{tot}}}\left( t \right) = n_i^{\mathrm{x}}\left( t \right) + n_i\left( t \right),i \in C: = \left\{ {{\mathrm{DN}},{\mathrm{DP}},{\mathrm{SP}}4,{\mathrm{cTEC}},{\mathrm{mTEC}}} \right\},$$

where *C* is the set of the cell types.

We describe the decrease in the irradiated cells by an exponential decay, which assumes that cells die at a constant rate *ω*_*i*_ after irradiation:

$$n_i^{\mathrm{x}}\left( t \right) = n_i^{\mathrm{x}}\left( 0 \right)e^{ - \omega _it}.$$

In the model, $n_i^{{\mathrm{tot}}}\left( 0 \right)$ represents the initial population size of type *i* cells and *p*_*i*_ is assumed to be the fraction of survived cells at *t* ≤ 0 as

$$\begin{array}{ll}n_i(t) =	 \left\{ {\begin{array}{*{20}{c}} {n_i^{{\mathrm{tot}}}\left( 0 \right),\quad t \,<\, 0} \\ {p_in_i^{{\mathrm{tot}}}(0),\quad t = 0} \end{array}} \right.,\\ n_i^{\mathrm{x}}\left( t \right) =	 \left\{ {\begin{array}{*{20}{l}}\qquad\quad\qquad {0},\quad\; {t\, <\, 0} \hfill \\ {\left( {1 - p_i} \right)n_i^{{\mathrm{tot}}}(0),\quad t = 0} \hfill \end{array}} \right..\end{array}$$

Given these initial conditions, the model of Eq. (1) was implemented on MATLAB (R2018a; The MathWorks, Natick, MA) and was numerically simulated by ‘dde23’ function or on Mathematica (version 11.2; Wolfram research, Champaign, Illinois) and simulated by ‘NDSolve’ function.

### Parameter estimation

In the parameter estimation, *ω*_*i*_, $n_i^{{\mathrm{tot}}}\left( 0 \right)$, *p*_*i*_, and all parameters appearing in Eq. (1) were simultaneously estimated. Parameters were estimated by minimizing the sum of the squares of difference between the logarithms of the observed data and simulated values of the model. Because the orders of the parameters are different, and this caused difficulty in the minimization, we decomposed the parameters as ${\mathbf{\theta }} = {\mathbf{\theta }}_{\mathrm{c}} \circ {\mathbf{\theta }}_{\mathrm{p}}$, where **θ**_c_ is a coefficient vector to estimate, **θ**_p_ is a constant vector of a power of 10, and $\circ$ denotes elementwise multiplication. For the observed time points ${\mathbf{t}}^ \ast = [t_1, \cdots ,t_m]$ and the corresponding data points *N*_*i*_(*t*_*j*_) for all $i \in C$, the estimated parameter set $\hat{\mathbf{\theta }}$ was obtained by solving

$$\widehat {\mathbf{\theta }}_{\mathrm{c}} =	\, {\mathrm{arg}}\,\min _{{\mathbf{\theta }}_{\mathrm{c}}}\sum_{j = 1}^{m} \sum_{i \in C} \left[ {\ln ( {n_i^{{\mathrm{{tot}}}}( {t_j,{\mathbf{\theta }}_{\mathrm{c}} \circ {\mathbf{\theta }}_{\mathrm{p}}} )}) - \ln ( {N_i( {t_j})})} \right]^2,\\ \quad \widehat {\mathbf{\theta }} =	\, \widehat {\mathbf{\theta }}_{\mathrm{c}} \circ {\mathbf{\theta }}_{\mathrm{p}}.$$

To solve this minimization, we used the ‘lsqnolin’ function in MATLAB Optimization Toolbox in which parameters were estimated by Trust Region Reflective method. The initial parameter values in the estimation were given, so that the result converges to moderate values considering the results of related previous works. The searching range of each parameter, except for *p*_*i*_, *r*_1_, and *r*_24_, was set between 10 and 0.1 times the initial value. Since *p*_*i*_, *r*_1_, and *r*_24_ represent fractions, their searching ranges were set between 0 and 1. The symbols, descriptions, and estimated values of the parameters are listed in Supplementary Table 1.

### Confidence interval by bootstrap

We calculated the confidence intervals of the estimated parameter values by a bootstrap method^51^.

First, for type *i* cells, we modeled the statistical variation of the data points using a Gaussian random variable $\varepsilon _i \sim {\cal{N}}(0,\sigma _i^2)$ with mean 0 and variance $\sigma _i^2$ as

$$\ln \left( {N_i\left( t \right)} \right) = \ln ( {n_i^{{\mathrm{tot}}}( {t,\widehat {\mathbf{\theta }}} )} ) + \varepsilon _i.$$

We estimated $\sigma _i^2$ by the sample variance as

$$\hat \sigma^2 _i = \frac{1}{{m - 1}}\mathop {\sum }\limits_{j = 1}^m [ {\ln N_i( {t_j}) - \ln n_i^{{\mathrm{tot}}}( {t_j,{\hat{\mathbf{\theta }}}})} ]^2.$$

We obtained the *k*th bootstrapped sample of the time point *t*_*j*_, $N_i^{b_k}( {t_j})$ by using a random number $\varepsilon _{i,j}^k \sim {\cal{N}}(0,\hat \sigma _i^2)$ as

$$\ln N_i^{b_k}\left( {t_j} \right) = \ln n_i^{{\mathrm{tot}}}\left( {t_j,{\hat{\mathbf{\theta }}}} \right) + \varepsilon _{i,j}^k.$$

The *k*th bootstrapped parameter set $\hat \theta ^{b_k}$ was obtained by solving the same optimization problem of the previous section by replacing the data with the *k*th bootstrapped sample $N_i^{b_k}( {{{t}}_{\mathrm{j}}})$ as

$${\hat{\mathbf{\theta}}}{}^{b_k} = \arg \min_{\mathbf{\theta }} \sum _{j = 1}^{m} \sum_{i \in C} [ {\ln ( {n_i^{{\mathrm{tot}}}( {t_j,{\mathbf{\theta }}} )} ) - \ln ( {N_i^{b_k}( {t_j} )})} ]^2.$$

The total number of the bootstrapped samples generated was *B* = 1000. The two-sided *α* × 100% confidence interval of the *l*th parameter was calculated as $[\hat \theta _l^{\left( {B(1 - \alpha )/2} \right)},\hat \theta _l^{(B\alpha /2)}]$, where $\hat \theta _l^{(x)}$ is the *x*th smallest value of the *l*th parameter obtained from the bootstrapped samples. We used *α* = 0.95. The confidence interval of each parameter is shown in Supplementary Table 1. A pairwise scatter plot of the bootstrap estimates is shown in Supplementary Fig. 3. The trajectories of cells obtained from 100 samples of the bootstrap parameter sets are shown in Fig. 2c.

### Detailed model of DN thymocytes

We additionally measured dynamic changes in the population sizes of DN1, DN2, DN3, and DN4 cells after irradiation.

To estimate the DN subpopulation dynamics in the original data (Fig. 1b), we utilized the DN subpopulations data as follows. First, we calculated the average proportions of the DN subpopulations at each time point. Subsequently, assuming that the dynamics of the DN subpopulation proportions were the same as the original data, we multiplied the number of DN cells from the original data with the calculated DN subpopulation proportions at each time point. At time points where we did not have corresponding DN subpopulation data (days 12 and 14), we used the average proportions of neighboring time points (days 11 and 13 for day 12, and days 13 and 15 for day 14).

Employing the obtained estimates of the DN subpopulation dynamics, we estimated the parameter values of the following detailed model of DN1, DN2, DN3, DN4, DP, and cTEC:

$$\frac{{{\mathrm{d}}n_{{\mathrm{DN}}1}}}{{{\mathrm{d}}t}} =	\, \phi _1 + (\delta _{{\mathrm{DN}}1} - \mu _{{\mathrm{DN}}1}n_{{\mathrm{cTEC}}}(t))n_{{\mathrm{DN}}1}(t),\\ \frac{{{\mathrm{d}}n_{{\mathrm{DN}}i}}}{{{\mathrm{d}}t}} =	\, \mu _{{\mathrm{DN}}i - 1}n_{{\mathrm{cTEC}}}\left( t \right)n_{{\mathrm{DN}}i - 1}\left( t \right) + \left( {\delta _{{\mathrm{DN}}i} + \mu _{{\mathrm{DN}}i}n_{{\mathrm{cTEC}}}\left( t \right)} \right)n_{{\mathrm{DN}}i}(t),\\ \frac{{{\mathrm{d}}n_{{\mathrm{DP}}}}}{{{\mathrm{d}}t}} =	\, r_{{\mathrm{DN}}4}\mu _{{\mathrm{DN}}4}n_{{\mathrm{cTEC}}}\left( t \right)n_{{\mathrm{DN}}4}\left( t \right) + \left\{ {\theta _2\left( {1 - \frac{{n_{{\mathrm{DP}}}\left( t \right)}}{{K_2}}} \right) - \mu _2n_{{\mathrm{cTEC}}}\left( {t - \tau _2} \right)} \right\}n_{{\mathrm{DP}}}(t),\\ \frac{{{\mathrm{d}}n_{{\mathrm{cTEC}}}}}{{{\mathrm{d}}t}} =	\, \phi _{\mathrm{c}} + \left( { - \delta _{\mathrm{c}} + \mathop {\sum }\limits_{j = 1}^4 \mu _{{\mathrm{cTEC}},j}n_{{\mathrm{DN}}j}\left( t \right)} \right)n_{{\mathrm{cTEC}}}\left( t \right),\\ \hskip-1.4pc{\mathrm{for}}\,i =	\, 2,3,4.$$

The parameter estimation procedure was the same as for the coarse-grained model. Because the detailed model has parameters common to the coarse-grained model, *ϕ*_1_ and the model parameters of DP and cTECs, except for $r_{{\mathrm{DN}}4}$, ${\mu} _{{\mathrm{DN}}4}$, and ${\mu} _{{\mathrm{cTEC}},i}$, we first fixed those parameter values to the estimates from the coarse-grained model and estimated the remaining parameter values. However, the detailed model with the estimated parameter values did not reproduce the DN1 dynamics (Supplementary Fig. 5). To obtain the parameter values capable of reproducing the dynamics of all cell types, we estimated parameter values including *ϕ*_1_ while other common parameter values were fixed (Fig. 4a and Supplementary Table. 2).

### Possible model (1): no self-suppression of mTEC

We constructed a model of mTEC without self-suppression (Fig. 5a, e) that had fewer parameters than the proposed model (Fig. 2a, b):

$$\frac{{{\mathrm{d}}n_{{\mathrm{mTEC}}}}}{{{\mathrm{d}}t}} = \phi _{\mathrm{m}} + \phi _{{\mathrm{m}}4}n_{{\mathrm{SP}}4}\left( t \right) - ( {\gamma _{{\mathrm{mp}}}n_{{\mathrm{DP}}}\left( {t - \tau _{\mathrm{m}}} \right) + \mu _{\mathrm{m}}} )n_{{\mathrm{mTEC}}}\left( t \right).$$

This model is less appropriate than the proposed one because the estimated value of the coefficient $\gamma _{{\mathrm{mp}}}n_{{\mathrm{DP}}}^ \ast + \mu _{\mathrm{m}}$ is so large that the mTECs die within a few hours.

### Possible model (2): regulation by DN to mTEC

We constructed the following model of mTECs with direct regulation by DN cells because the temporal peaks of their population sizes coincided in the data (Fig. 5b, f):

$$\frac{{{\mathrm{d}}n_{{\mathrm{mTEC}}}}}{{{\mathrm{d}}t}} = \phi _{{\mathrm{mn}}}n_{{\mathrm{DN}}}\left( t \right) + \phi _{{\mathrm{m}}4}n_{{\mathrm{SP}}4}\left( t \right) - \mu _{\mathrm{m}}n_{{\mathrm{mTEC}}}\left( t \right).$$

We rejected this model because we have no evidence of direct interaction between DN thymocytes and mTECs, which are located in different regions of a thymus.

### Possible model (3): increase of DP by cTEC

We constructed a model of the DP cells in which cTECs promote the increase in the DP population size by assuming that cTECs either induce DP proliferation or rescue DP thymocytes from apoptosis in positive selection (Fig. 5c, g):

$$\frac{{{\mathrm{d}}n_{{\mathrm{DP}}}}}{{{\mathrm{d}}t}} = \mu _1r_1n_{{\mathrm{cTEC}}}\left( t \right)n_{{\mathrm{DN}}}\left( t \right) + r_2n_{{\mathrm{DP}}}\left( t \right)\left( {1 - \frac{{n_{{\mathrm{DP}}}\left( t \right)}}{{K_2}}} \right) + \mu _2n_{{\mathrm{cTEC}}}\left( t \right)n_{{\mathrm{DP}}}\left( t \right).$$

This model was determined inappropriate because the estimated values of the coefficients $r_2(1 - n_{{\mathrm{DP}}}^ \ast /K_2)$and $\mu _2n_{{\mathrm{cTEC}}}^ \ast$ were so large that self-replication and apoptosis occurred within a few hours.

### Possible model (4): regulation by DP to cTEC

We constructed a model of cTECs with a regulation by DP thymocytes (Fig. 5d, h) because the depletion of DP thymocytes was reported to induce recovery both of mTECs and cTECs^5^:

$$\frac{{{\mathrm{d}}n_{{\mathrm{cTEC}}}}}{{{\mathrm{d}}t}} = \phi _{\mathrm{c}} + \left( { - \delta _{\mathrm{c}} + \mu _{\mathrm{c}}n_{{\mathrm{DN}}}\left( t \right) - \gamma _{\mathrm{c}}n_{{\mathrm{DP}}}\left( {t - \tau _{\mathrm{m}}} \right)} \right)n_{{\mathrm{cTEC}}}\left( t \right).$$

We found that the assumed effect of DP cells on cTECs was negligible because the substitution of 0 to *γ*_*c*_ did not change the dynamics after the parameter estimation. Thus, we did not adopt this additional interaction.

### Reporting Summary

Further information on research design is available in the Nature Research Reporting Summary linked to this article.

## Supplementary information

Supplementary Information

Description of Additional Supplementary Files

Supplementary Data 1

Reporting Summary

## Data Availability

Source data of thymocyte and TEC population sizes are available as Supplementary Data 1. All other relevant data, if any, are available from the authors.

## Code availability

The utilized computer code and parameters of the models are available at https://github.com/Q-bio-at-IIS/Kaneko2019CommBiol.
