# Supplementary material for: Quantitative analysis reveals reciprocal regulations underlying recovery dynamics of thymocytes and thymic environment in mice
Source: Commun Biol. 2019 Nov 29;2:444. doi: 10.1038/s42003-019-0688-8 (PMC6884561; doi:10.1038/s42003-019-0688-8)
Supplement: Supplementary file 1 — Supplementary Information [file 42003_2019_688_MOESM1_ESM.docx]

**Supplementary Information**

**Supplementary Figures**


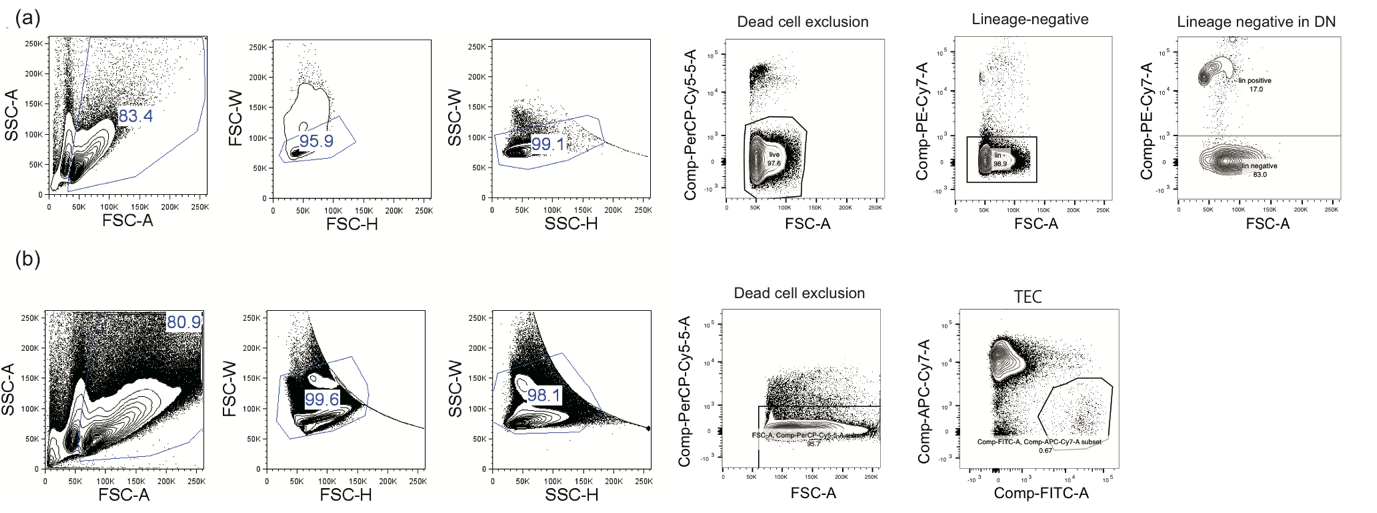


Supplementary Figure 1: (a) Gating strategy for thymocytes. (b) Gating strategy for TECs


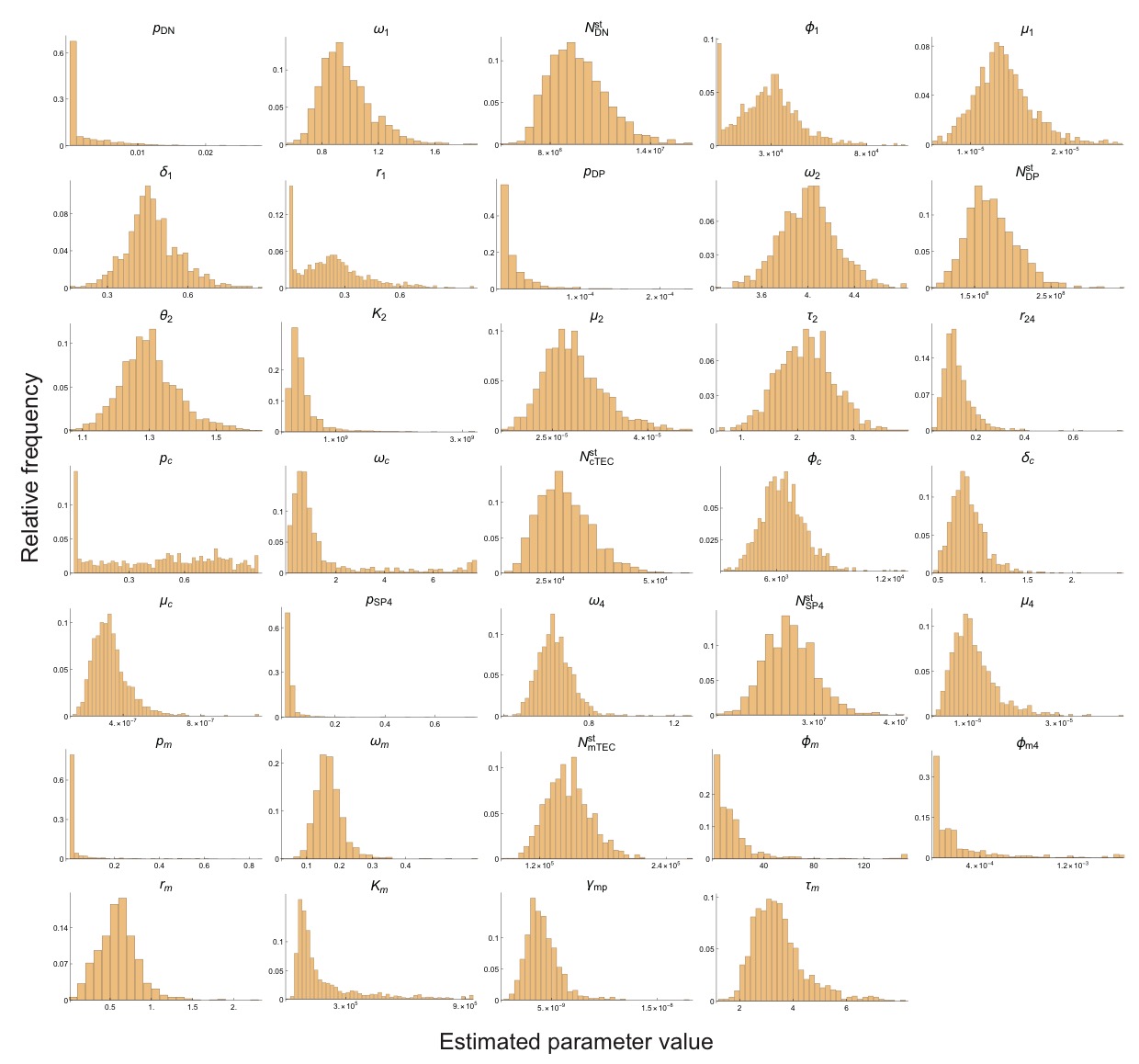
Supplementary Figure 2: Histograms of the parameter values obtained by the bootstrap estimation.


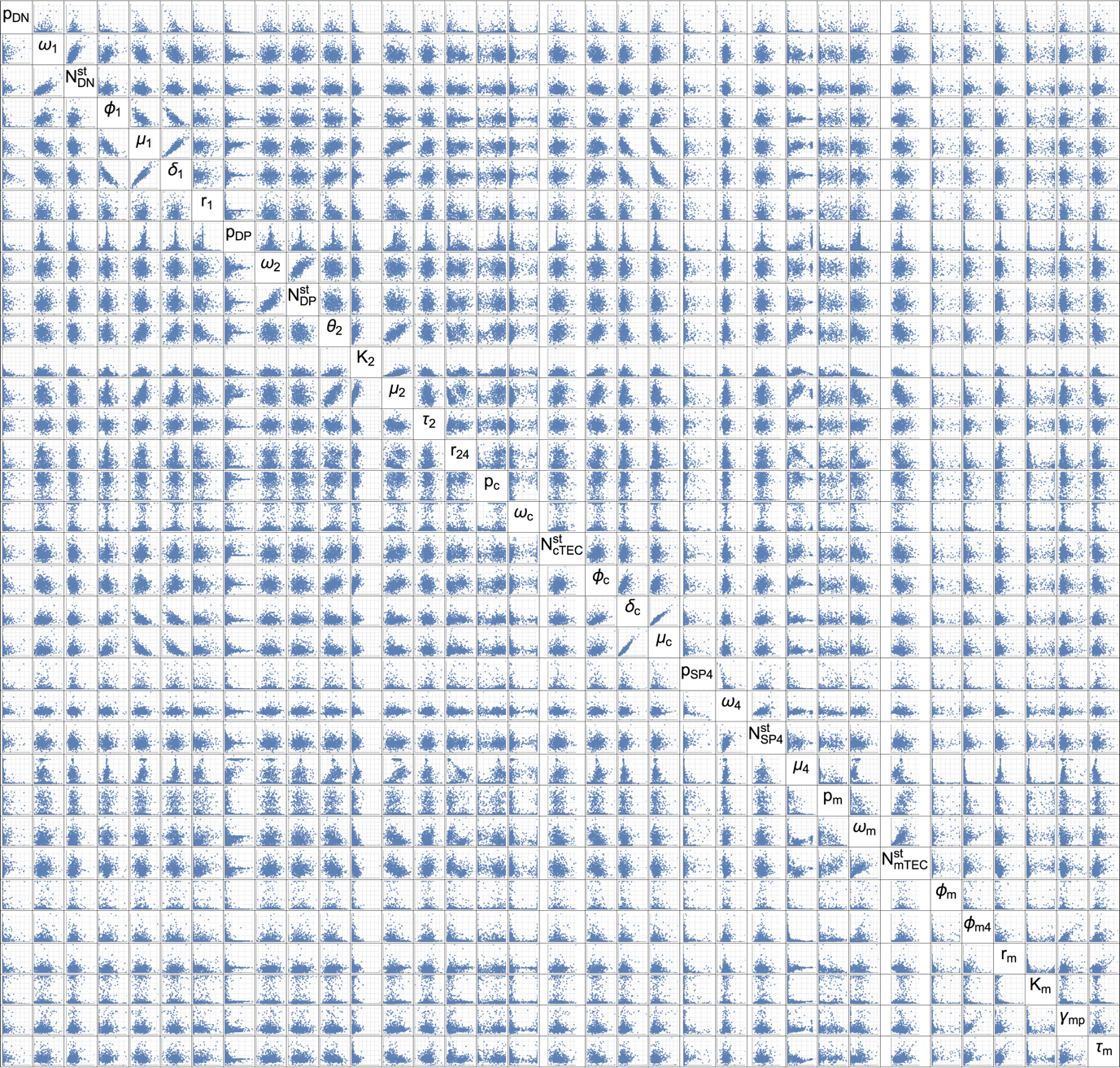


Supplementary Figure 3: Two-dimensional scatter plots of the parameter values obtained by the bootstrap estimation. The plot range of each parameter is set to be the same as that in Supplementary Fig. 2.


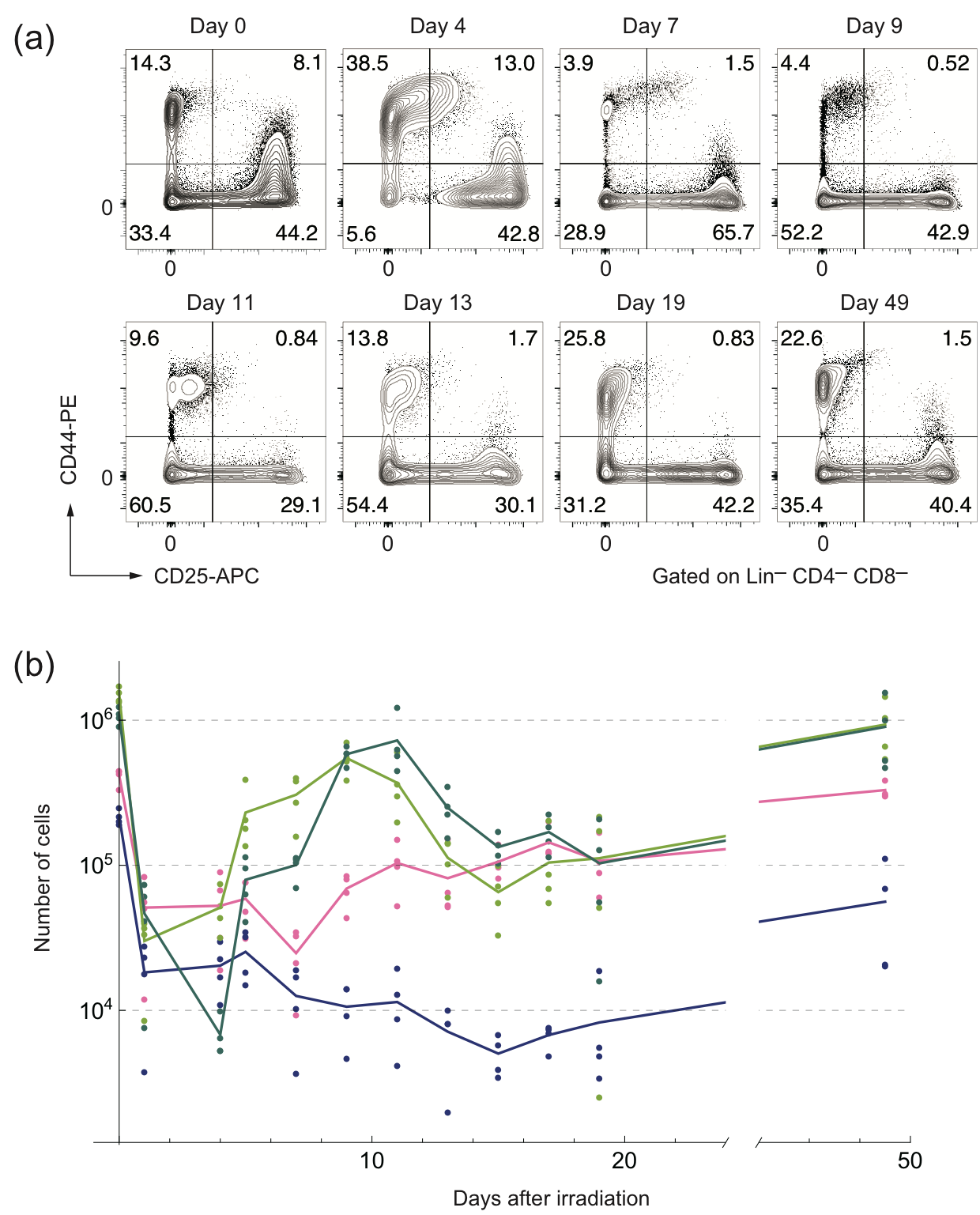


Supplementary Figure 4: (a) Typical flow cytometric profiles of DN thymocytes after the sub-lethal dose radiation. Lineage marker-negative, CD4-negative, CD8-negative thymocytes were analyzed by staining with anti-CD25 and anti-CD44. Percentage of each fraction is shown in the panels. (b) The trajectories of the counts of the DN1 (pink), DN2 (blue), DN3 (light green), DN4 (green) thymocytes after the irradiation. Points correspond to the experimental counts of the cells, and the solid curves are linear interpolations of the average counts at each time point. (n=4 at each time point)

Supplementary Figure 5: Trajectories of the detailed model as a result of fitting to the experimentally obtained dynamics of DN subpopulations while keeping the values of the parameters common to the coarse-grained model fixed. DN1 : pink, DN2 : blue, DN3 : light green, DN4 : green.

**Supplementary**  **Tables**

Supplementary Table 1. Estimated parameter values in the coarse-grained model. (CI : confidence interval). We should note that this is the first systematic estimate of the parameters related to the dynamics of TECs. These parameters should be verified by independent researches.

| Symbol | Description | Value | CI |
| --- | --- | --- | --- |
| p_DN_ | Proportion of normal cells in the initial number of DN | 1.71 × 10^-5^ | [1.39 × 10^-7^, 1.18 × 10^-2^] |
| ω_DN_ | Death rate of irradiated DN | 9.24 × 10^-1^ | [7.04 × 10^-1^, 1.40 × 10^0^] |
| n_DN_^tot^(0) | Initial value for the number of DN | 9.29 × 10^6^ | [6.89 × 10^6^, 1.38 × 10^7^] |
| φ_1_ | Inflow to DN | 3.34 × 10^4^ | [3.34 × 10^3^, 6.56 × 10^4^] |
| μ_1_ | Negative regulation by cTEC to DN | 1.35 × 10^-5^ | [8.08 × 10^-6^, 2.11 × 10^-5^] |
| δ_1_ | Intrinsic proliferation rate of DN | 4.69 × 10^-1^ | [2.84 × 10^-1^, 6.94 × 10^-1^] |
| r_1_ | Proportion of differentiation to DP in regulation by cTEC | 2.95 × 10^-1^ | [1.57 × 10^-5^, 6.91 × 10^-1^] |
| p_DP_ | Proportion of normal cells in the initial number of DP | 6.87 × 10^-6^ | [4.53 × 10^-9^, 8.39 × 10^-5^] |
| ω_DP_ | Death rate of irradiated DP | 4.00 × 10^0^ | [3.51 × 10^0^, 4.51 × 10^0^] |
| n_DP_^tot^(0) | Initial value for the number of DP | 1.70 × 10^8^ | [1.18 × 10^8^, 2.47 × 10^8^] |
| θ_2_ | Proliferation rate of DP | 1.31 × 10^0^ | [1.14 × 10^0^, 1.51 × 10^0^] |
| K_2_ | Carrying capacity of DP | 4.23 × 10^8^ | [2.42 × 10^8^, 1.39 × 10^9^] |
| μ_2_ | Negative regulation rate by cTEC to DP | 2.85 × 10^-5^ | [2.06 × 10^-5^, 4.02 × 10^-5^] |
| τ_2_ | Delay of cTEC regulating DP | 2.12 × 10^0^ | [1.17 × 10^0^, 3.12 × 10^0^] |
| r_24_ | Proportion of differentiation to CD4+SP in regulation by cTEC | 1.03 × 10^-1^ | [4.94 × 10^-2^, 2.96 × 10^-1^] |
| p_cTEC_ | Proportion of normal cells in the initial number of cTEC | 7.65 × 10^-1^ | [1.10 × 10^-4^, 9.83 × 10^-1^] |
| ω_cTEC_ | Death rate of irradiated cTEC | 7.67 × 10^-1^ | [9.28 × 10^-2^, 7.58 × 10^0^] |
| n_cTEC_^tot^(0) | Initial value for the number of cTEC | 2.71 × 10^4^ | [1.87 × 10^4^, 4.34 × 10^4^] |
| φ_c_ | Inflow to cTEC | 6.47 × 10^3^ | [4.26 × 10^3^, 8.75 × 10^3^] |
| δ_c_ | Death rate of cTEC | 7.94 × 10^-1^ | [5.39 × 10^-1^, 1.29 × 10^0^] |
| μ_c_ | Positive regulation by DN to cTEC | 3.13 × 10^-7^ | [1.96 × 10^-7^, 5.73 × 10^-7^] |
| p_SP4_ | Proportion of normal cells in the initial number of CD4+SP | 1.47 × 10^-2^ | [8.22 × 10^-4^, 1.11 × 10^-1^] |
| ω_SP4_ | Death rate of irradiated CD4+SP | 6.31 × 10^-1^ | [5.04 × 10^-1^, 8.28 × 10^-1^] |
| n_SP4_^tot^(0) | Initial value for the number of CD4+SP | 2.66 × 10^7^ | [2.16 × 10^7^, 3.38 × 10^7^] |
| μ_4_ | Negative regulation by mTEC to CD4+SP | 9.85 × 10^-6^ | [4.70 × 10^-6^, 2.47 × 10^-5^] |
| p_mTEC_ | Proportion of normal cells in the initial number of mTEC | 3.15 × 10^-6^ | [5.53 × 10^-8^, 4.39 × 10^-1^] |
| ω_mTEC_ | Death rate of irradiated mTEC | 1.60 × 10^-1^ | [9.97 × 10^-2^, 2.79 × 10^-1^] |
| n_mTEC_^tot^(0) | Initial value for the number of mTEC | 1.44 × 10^5^ | [1.10 × 10^5^, 1.93 × 10^5^] |
| φ_m_ | Constant inflow to mTEC | 1.52 × 10^1^ | [1.52 × 10^0^, 1.18 × 10^2^] |
| φ_m4_ | Inflow to mTEC regulated by CD4+SP | 1.60 × 10^-4^ | [1.60 × 10^-5^, 1.53 × 10^-3^] |
| r_m_ | Proliferation rate of mTEC | 6.10 × 10^-1^ | [1.67 × 10^-1^, 1.16 × 10^0^] |
| K_m_ | Carrying capacity of mTEC | 9.22 × 10^4^ | [4.94 × 10^4^, 7.41 × 10^5^] |
| γ_mp_ | Negative regulation rate by DP to mTEC | 3.50 × 10^-9^ | [1.76 × 10^-9^, 8.18 × 10^-9^] |
| τ_m_ | Delay of DP regulating mTEC | 3.27 × 10^0^ | [2.09 × 10^0^, 6.19 × 10^0^] |

Supplementary Table 2. Estimated parameter values in the detailed model.

| Symbol | Description | Value |
| --- | --- | --- |
| φ_1_ | Inflow to DN | 5.65 × 10^1^ |
| δ­­­­_DN1­_ | Intrinsic proliferation rate of DN1 | 5.42 × 10^-1^ |
| δ_DN2_ | Intrinsic proliferation rate of DN2 | 3.26 × 10^-5^ |
| δ_DN3_ | Intrinsic proliferation rate of DN3 | 7.38 × 10^-1^ |
| δ_DN4_ | Intrinsic proliferation rate of DN4 | 4.14 × 10^-1^ |
| μ_DN1_ | Negative regulation by cTEC to DN1 | 1.32 × 10^-5^ |
| μ_DN2_ | Negative regulation by cTEC to DN2 | 1.25 × 10^-4^ |
| μ_DN3_ | Negative regulation by cTEC to DN3 | 3.30 × 10^-5^ |
| μ_DN4_ | Negative regulation by cTEC to DN4 | 3.39 × 10^-5^ |
| μ_cTEC1_ | Positive regulation by DN1 to cTEC | 2.59 × 10^-7^ |
| μ_cTEC2_ | Positive regulation by DN2 to cTEC | 1.06 × 10^-8^ |
| μ_cTEC3_ | Positive regulation by DN3 to cTEC | 2.80 × 10^-9^ |
| μ_cTEC4_ | Positive regulation by DN4 to cTEC | 5.33 × 10^-7^ |
| n_DN1_^tot^(0) | Initial value for the number of DN1 | 1.80 × 10^6^ |
| n_DN2_^tot^(0) | Initial value for the number of DN2 | 7.09 × 10^5^ |
| n_DN3_^tot^(0) | Initial value for the number of DN3 | 2.86 × 10^6^ |
| n_DN4_^tot^(0) | Initial value for the number of DN4 | 3.84 × 10^6^ |
| p_DN1_ | Proportion of normal cells in the initial number of DN1 | 2.30 × 10^-3^ |
| p_DN2_ | Proportion of normal cells in the initial number of DN2 | 2.37 × 10^-2^ |
| p_DN3_ | Proportion of normal cells in the initial number of DN3 | 4.08 × 10^-4^ |
| p_DN4_ | Proportion of normal cells in the initial number of DN4 | 2.47 × 10^-5^ |
| ω_DN1_ | Death rate of irradiated DN1 | 6.24 × 10^-1^ |
| ω_DN2_ | Death rate of irradiated DN2 | 5.14 × 10^-1^ |
| ω_DN3_ | Death rate of irradiated DN3 | 8.95 × 10^-1^ |
| ω_DN4_ | Death rate of irradiated DN4 | 1.44 × 10^0^ |
| r_DN4_ | Proportion of differentiation from DN4 to DP in regulation by cTEC | 6.67 × 10^-1^ |
