# Supplementary material for: Quantitative analysis reveals reciprocal regulations underlying recovery dynamics of thymocytes and thymic environment in mice
Source: Commun Biol. 2019 Nov 29;2:444. doi: 10.1038/s42003-019-0688-8 (PMC6884561; doi:10.1038/s42003-019-0688-8)
Supplement: Supplementary file 2 — Description of Additional Supplementary Files [file 42003_2019_688_MOESM2_ESM.docx]

**Description of Additional Supplementary Files**

**Supplementary Data 1:** Source data file
