# Supplementary material for: Quantitative analysis reveals reciprocal regulations underlying recovery dynamics of thymocytes and thymic environment in mice
Source: Commun Biol. 2019 Nov 29;2:444. doi: 10.1038/s42003-019-0688-8 (PMC6884561; doi:10.1038/s42003-019-0688-8)
Supplement: Supplementary file 4 — Reporting Summary [file 42003_2019_688_MOESM4_ESM.pdf]

nature research

Corresponding author(s): Taishin Akiyama and Tetsuya J. Kobayashi

Last updated by author(s): Sep. 18, 2019

Reporting Summary

Nature Research wishes to improve the reproducibility of the work that we publish. This form provides structure for consistency and transparency in reporting. For further information on Nature Research policies, see Authors & Referees and the Editorial Policy Checklist.

Statistics

For all statistical analyses, confirm that the following items are present in the figure legend, table legend, main text, or Methods section.

n/a	Confirmed	
☐	☒	The exact sample size (*n*) for each experimental group/condition, given as a discrete number and unit of measurement
☐	☒	A statement on whether measurements were taken from distinct samples or whether the same sample was measured repeatedly
☐	☒	The statistical test(s) used AND whether they are one- or two-sided<br>*Only common tests should be described solely by name; describe more complex techniques in the Methods section.*
☒	☐	A description of all covariates tested
☐	☒	A description of any assumptions or corrections, such as tests of normality and adjustment for multiple comparisons
☐	☒	A full description of the statistical parameters including central tendency (e.g. means) or other basic estimates (e.g. regression coefficient) AND variation (e.g. standard deviation) or associated estimates of uncertainty (e.g. confidence intervals)
☒	☐	For null hypothesis testing, the test statistic (e.g. *F*, *t*, *r*) with confidence intervals, effect sizes, degrees of freedom and *P* value noted<br>*Give P values as exact values whenever suitable.*
☒	☐	For Bayesian analysis, information on the choice of priors and Markov chain Monte Carlo settings
☒	☐	For hierarchical and complex designs, identification of the appropriate level for tests and full reporting of outcomes
☒	☐	Estimates of effect sizes (e.g. Cohen's *d*, Pearson's *r*), indicating how they were calculated

Our web collection on statistics for biologists contains articles on many of the points above.

Software and code

Policy information about availability of computer code

Data collection	Canto II, Aria II or III, FACS Diva ver.8;
Data analysis	Flowjo 10, MATLAB (R2016b; The MathWorks, Natick, MA), Mathematica (version 11.2; Wolfram research, Champaign, Illinois)

For manuscripts utilizing custom algorithms or software that are central to the research but not yet described in published literature, software must be made available to editors/reviewers. We strongly encourage code deposition in a community repository (e.g. GitHub). See the Nature Research guidelines for submitting code & software for further information.

Data

Policy information about availability of data

All manuscripts must include a data availability statement. This statement should provide the following information, where applicable:

- Accession codes, unique identifiers, or web links for publicly available datasets
- A list of figures that have associated raw data
- A description of any restrictions on data availability

All data is available as supplementary data. All codes are available at https://github.com/Q-bio-at-IIS/Kaneko2019CommBiol.

Field-specific reporting

Please select the one below that is the best fit for your research. If you are not sure, read the appropriate sections before making your selection.

☒ Life sciences ☐ Behavioural & social sciences ☐ Ecological, evolutionary & environmental sciences

For a reference copy of the document with all sections, see nature.com/documents/nr-reporting-summary-flat.pdf

Life sciences study design

All studies must disclose on these points even when the disclosure is negative.

Sample size	No statistical methods were done to predetermine sample size.
Data exclusions	There is no data exclusion.
Replication	All samples were done in biological and technical replicates. All experiments were replicated at least two times. All replications were similar results.
Randomization	No randomization was done.
Blinding	No blind test was done

Reporting for specific materials, systems and methods

We require information from authors about some types of materials, experimental systems and methods used in many studies. Here, indicate whether each material, system or method listed is relevant to your study. If you are not sure if a list item applies to your research, read the appropriate section before selecting a response.

Materials & experimental systems

n/a	Involved in the study	
☐	☒	Antibodies
☒	☐	Eukaryotic cell lines
☒	☐	Palaeontology
☐	☒	Animals and other organisms
☒	☐	Human research participants
☒	☐	Clinical data

Methods

n/a	Involved in the study	
☒	☐	ChIP-seq
☐	☒	Flow cytometry
☒	☐	MRI-based neuroimaging

Antibodies

Antibodies used	PECy7-anti-CD4 (clone RAM4-4, used as x200 dilution), FITC-anti-CD4 (clone RAM4-4, x200 dilution), APCCy7-anti-CD8 (clone 53-6.7, x200), APCCy7-anti-CD45 (clone 30 F-11, x200), APCCy7-anti-TER119 (clone TER-119, x200), FITC-anti-EpCAM (BioLegend, clone G8.8, x400), PE-anti-CD80(clone 16-10A1, x400), Biotin-anti-mouse Ly-6G/Ly-6C(Gr-1) (x400),, Biotin anti-mouse/human CD45R/B220 (x400), Biotin anti-mouse TER-119/Erythroid cells (clone TER-119, x400), Biotin conjugated anti-mouse CD11b (x400), PE anti-mouse/human CD44 (clone IM7, x400), APC anti-mouse CD25 (clonePC61, x400), Streptavidin PE-Cyanine7 (x400), and Streptavidin-PECy7 (x400) were purchased from Biolegnd. UEA-biotin (x400) was from Vector laboratories (Burlingame, CA).
Validation	Antibodies were validated in the companies from which they were purchased

Animals and other organisms

Policy information about studies involving animals; ARRIVE guidelines recommended for reporting animal research

Laboratory animals	Seven week-old female Balb/cA mice were used in this study.
Wild animals	No wild animals were used.
Field-collected samples	No field collected samples were used.
Ethics oversight	All mice were handled in accordance with the Guidelines for the Institutional Animal Care and Use Committee (IACUC) of RIKEN Yokohama Branch.

Note that full information on the approval of the study protocol must also be provided in the manuscript.

Flow Cytometry

Plots

Confirm that:

☒ The axis labels state the marker and fluorochrome used (e.g. CD4-FITC).

☒ The axis scales are clearly visible. Include numbers along axes only for bottom left plot of group (a 'group' is an analysis of identical markers).

☒ All plots are contour plots with outliers or pseudocolor plots.

☒ A numerical value for number of cells or percentage (with statistics) is provided.

Methodology

Sample preparation	Each thymus was cut and gently agitated in 2 ml of RPMI-1640 (Sigma-Aldrich, St. Louis, MO, U.S.A.) to release thymocytes for the flow cytometric analysis. The rest of the thymic tissue was digested using Liberase in RPMI1640 (Wako) at 37 degree for 30 min. The thymic stroma rich-fraction was analyzed by flow cytometry to detect the TEC populations.
Instrument	FACS Aria and Canto II (BD).
Software	Flowjo
Cell population abundance	Not available
Gating strategy	Shown in Figure 1 and supplementary figure

☒ Tick this box to confirm that a figure exemplifying the gating strategy is provided in the Supplementary Information.
